# Financial vulnerability of the elderly population undergoing cataract surgery within a distributed eye care delivery system in India (2011–2022): a multicentre, retrospective cohort study

**DOI:** 10.1016/j.lansea.2025.100640

**Published:** 2025-07-23

**Authors:** Brijesh Takkar, Ragukumar Venugopal, Mehul Mehta, Anthony Vipin Das, Varsha Rathi, Rohit Khanna, Gudlavalleti V.S. Murthy, Hemendra Kumar Vaishnav, Brijesh Kashyap, Chirantan Chatterjee, Raja Narayanan

**Affiliations:** a Indian Health Outcomes, Public Health, and Economics Research Center, L V Prasad Eye Institute, Hyderabad, Telangana, India; b Anant Bajaj Retina Institute, L V Prasad Eye Institute, Hyderabad, Telangana, India; c Department of Ophthalmology, Harvard Medical School, Boston, MA, USA; d Department of eyeSmart EMR & AEye, L V Prasad Eye Institute, Hyderabad, Telangana, India; e Allen Foster Community Eye Health Research Centre, Gullapalli Pratibha Rao International Center for Advancement of Rural Eye Care, LV Prasad Eye Institute, Hyderabad, Telangana, India; f Pragyaan Sustainable Health Outcomes Foundation, India; g Department of Economics, University of Sussex, UK

**Keywords:** Eye care for elderly, Geriatric eye care, Health insurance for old people, Eye care policy, Out-of-pocket expenditures, Ophthalmology, Cataract, Insurance uptake

## Abstract

**Background:**

The health burden of geriatric eye care is expected to rise, yet medical insurance uptake among the elderly population remains low. There is minimal evidence regarding insurance uptake for eye care among the elderly population in the Southeast Asia region. We explored insurance uptake and its impact on visual outcomes among the elderly population who visited an eye care system distributed across four Indian states.

**Methods:**

In this retrospective cohort study, we used a browser-based proprietary, Hospital Information Management System (HIMSS) stage six, electronic medical record (EMR) system. Datasets were collected from new patients who visited the four tertiary centres with linked primary and secondary centres of our pyramidal health system (L V Prasad Eye Institute [India]) distributed in four Indian states, Andhra Pradesh, Odisha, Telangana, and Karnataka, between August 2011 and December 2022. The electronic medical records of 38,387 patients aged >70 years who underwent cataract surgery were included (45.5% were females [17,471]). Individuals treated with fully subsidised care were excluded. Data on age, health insurance uptake, type of health insurance (government or private), and mode of payment for cataract surgery were collected. Factors impacting insurance uptake and visual outcomes were studied using logistic regression analysis.

**Findings:**

Insurance uptake declined from 17.5% among people aged 70 years to less than 10% among those aged >85 years. Private insurance uptake declined from 13.3% among people aged 70 years to 4.7% among those aged 90 years, while publicly funded insurance remained between 3.3% and 4.2%. Insurance uptake increased during 2018–2022 compared to 2011–2017 (20.61% vs. 10.65%; p < 0.001). A higher proportion of males had insurance uptake compared to females. Median waiting times for surgery among patients with government versus private insurance were 18 and 11 days, respectively. Among patients aged >80 years, surgical outcomes for those without insurance were worse than for those with insurance.

**Interpretation:**

Insurance uptake declined dramatically in patients aged above 80 years and was associated with poorer visual outcomes following cataract surgery, as the insurance uptake may impact the quality of eye care received. Policy changes are needed to increase insurance uptake for eye care in this population.

**Funding:**

10.13039/501100009053DBT Wellcome Trust India Alliance, Clinical Research Centre Grant IA/CRC/19/1/610010; 10.13039/501100005809Hyderabad Eye Research Foundation (HERF).

**Research in context**

**Evidence before this study**

Globally, the proportion of individuals aged over 60 years is estimated to double in the next quarter-century, reaching one-fifth of the total population. The growing demand for geriatric eye care, coupled with the increasing economic burden faced by this population, presents a significant health equity issue. Current evidence indicates variable levels of medical insurance coverage among elderly populations worldwide. In India, senile cataract is the leading cause of blindness among this age group and is treated through well-standardised eye surgery systems.

**Added value of this study**

The availability of adequate health insurance coverage is a key determinant of the financial vulnerability of the geriatric population. This study offers a detailed evaluation of insurance uptake trends among a large proportion of India's geriatric population, using cataract surgery as a proxy for eye care. Our findings show that insurance uptake among the oldest and most vulnerable segments of the population is significantly inadequate in India. This is mainly due to insufficient universal coverage for the elderly in current government schemes and difficulties in obtaining private insurance. In this study, we provide robust evidence supporting the expansion of insurance coverage for the geriatric population, based on its association with their eye care needs. We also show that some elderly patients prefer government insurance programmes, even when offered a full subsidy by the care provider.

**Implications of all the available evidence**

Insurance coverage in general, and publicly funded schemes in particular, must be strengthened to include vision and eye care for elderly populations, especially those aged 80 years and above. This has a direct impact on patients' visual disability and can be a critical step towards achieving truly universal eye health coverage and visual health equity.

## Introduction

The WHO estimates indicate that the proportion of elderly population (individuals aged over 60 years) is expected to rise from 12% to 22% by 2050.^1^ This vulnerable population is also responsible for a significant percentage of the global burden of visual disability and eye diseases.^2^ In India, the demographic of individuals >65 years is set to increase 2.5 fold, from its current proportion of 8% of the total population to 20% by 2050. Blindness is prevalent in this population, with over 45% of people with age >60 years suffering from some form of visual impairment.^3^ Cataracts, the most common cause of avoidable blindness globally, are also prevalent in this population. Despite a global increase in the number of cataract surgeries, evidence, including that from India, indicates increasing prevalence rates of visual impairment and blindness due to cataracts in elderly populations. This has been reported in both population-based and hospital records-based studies.^4^, ^5^, ^6^, ^7^, ^8^, ^9^, ^10^, ^11^, ^12^, ^13^, ^14^ These health issues in the elderly populations are a significant economic burden for governments and societies. Therefore, the majority of the elderly people are opting for publicly funded health insurance schemes, even in low- and middle-income countries (LMICs).^15^ When coupled with government incentives such as rebates, the private insurance systems have seen a modest increase in the utilisation of health care by the elderly population in high-income countries.^16^ As a result, the efforts to improve insurance coverage for elderly populations through administrative and governmental reforms have been successful.^17^

**Supplementary Fig. S1:**
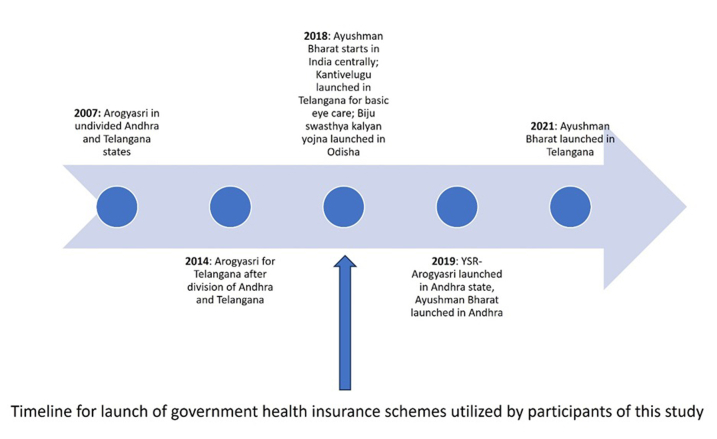
**Timeline showing government health insurance schemes utilised by the study population; Arrow indicates the “shock” induced by launch of three major schemes simultaneously in 2018; the *Biju Swasthya Kalyan Yojana* (BSKY) scheme is incidentally also the scheme with the highest penetration in the study population for eye and cataract care**.

The insurance system in India can be divided into government-funded health insurance (state and central); group health insurance (corporate) and individual health insurance. Health insurance in India covers mainly hospitalisation expenses, rather than outpatient or drug costs. In India, although insurance regulations came into being in 1999, a major surge in public-funded insurance has occurred only in the last 10–15 years while private health insurance existed even before.^18^ This includes a major nationwide centrally-implemented national health insurance system (*Ayushman Bharat*) that was launched in 2018 (Supplementary Fig. S1).^19^ This publicly funded system aims to benefit individuals belonging to lower socio-economic strata identified through census data, providing insurance benefits up to INR 500,000 (around US$ 5835 [as of June 26, 2025]) without any age limits. Among all age groups, those over 60 years showed the maximum utilisation of the scheme, highlighting the need for care in the elderly population.^19^ However, the National Family Health Survey-5 (2019–2021) revealed that only 11.50% of those aged over 60 years have any form of insurance coverage, indicating insufficient protection for this vulnerable age group.^20^ To our knowledge, no data exists on expenditure choices or coverage preferences made by the elderly population when it comes to eye care.

Since cataracts are responsible for the greatest eye care-related vision burden in the elderly population,^4^, ^5^, ^6^, ^7^, ^8^, ^9^, ^10^, ^11^, ^12^, ^13^, ^14^^,^^21^^,^^22^ an analysis of large data sets on surgery for cataract in the elderly population (aged >70 years) may offer a unique opportunity to study the insurance usage profile for eye care in this population. In the *Ayushman Bharat* scheme, over 98% of ophthalmology reimbursements are attributed to cataract surgical procedures, highlighting the significant burden of cataract procedures and cataract related visual impairment. In this study, we utilised structured, high-fidelity, electronic medical records (EMR) data from a large, distributed eye care network in India, that caters to patients in a three-tier referral pyramidal structure. Using these EMR-generated data sets, we studied the associations of demographics and major government schemes with patterns of insurance coverage and impact on visual outcomes. Based on these analyses, policy recommendations to prioritise the elderly population for eye health insurance may be considered.

## Methods

### Study design

For data collection, this hospital-based retrospective study utilised a browser-based proprietary, HIMSS (Hospital Information Management System) stage six, electronic medical record (EMR) system developed specifically for ophthalmology. Datasets were collected from new patients who visited the four tertiary centres with linked primary and secondary centres of our pyramidal health system between August 2011 and December 2022. All patient data in the EMR system were entered by uniformly trained ophthalmic personnel using a standardised template and were reviewed by the attending ophthalmologist. The study adhered to the tenets of the Declaration of Helsinki and institutional ethics clearance was obtained from the institute review board of the LV Prasad Eye Institute (Approval Number: LEC-BHR-R-10-22-954).

### Study population

The study cohort comprised of individuals aged >70 years (as corroborated via the government-provided unique identification card wherever available).^23^ We included people presenting to our eye care system, which is distributed in four states of India: Andhra Pradesh, Odisha, Telangana, and Karnataka.^24^

#### Inclusion criteria

Patients aged >70 years who had undergone any type of cataract surgery procedure (phacoemulsification, small incision cataract surgery [SICS], extracapsular cataract extraction [ECCE], and intracapsular cataract extraction [ICCE] with intraocular lens [IOL] implantation) were included. We included patients who either paid out-of-pocket (OOP) or had health insurance coverage. For patients who had both eyes operated for cataract, details of only the first eye were included for analysis.

#### Exclusion criteria

Patients for whom the payment mechanisms could not be clearly identified were excluded. Fully subsidised (FS) patients, who were provided free eye treatment by the institute, were excluded from the study. The FS patients were excluded as our objective was to understand the financial vulnerability of the elderly.

### Data retrieval and processing

The EMR was used to label payment categories for all patients.^24^ Data from a total of 92,791 surgical records were retrieved and sorted into a Microsoft Excel (Version 16.40) spreadsheet. Following screening of patients who met the study criteria, a total of 38,387 patients' data were included in the study. Included patients (aged 70–100 years) were systematically categorised into five-year bands, with those aged 70–74 years categorised as ‘youngest old’, and those aged 90 years and above were categorised as ‘oldest old’ (Tables 1 and 2).^25^

**Table 1:** Distribution of insurance users and out-of-pocket (OOP) payers amongst the categories of study population.

Variables	Insurance user n (%)	OOP n (%)
Gender		
Male—20,916	3998 (19.11)	16,918 (80.89)
Female—17,471	2172 (12.43)	15,299 (87.57)
Age		
70–74 years—22,681	3974 (17.52)	18,707 (82.48)
75–79 years—10,357	1651 (15.94)	8706 (84.06)
80–84 years—3914	426 (10.88)	3488 (89.12)
85–89 years—1165	99 (8.50)	1066 (91.50)
90–100 years—270	20 (7.41)	250 (92.59)
District-wise distribution		
Rural—18,904	1831 (9.69)	17,073 (90.31)
Urban—14,845	2626 (17.69)	12,219 (82.31)
Metropolitan—4638	1713 (36.93)	2925 (63.07)
Socioeconomic status		
Lower middle class—35,134	5246 (14.93)	29,888 (85.07)
Lower class—189	189 (100.0)	0 (0)
Upper middle class—2099	449 (21.39)	1650 (78.61)
Upper class—965	286 (29.64)	679 (70.36)
Systemic condition		
Diabetes or hypertension—10,395	2095 (20.15)	8300 (79.85)
Occupation		
Working—2072	451 (21.77)	1621 (78.23)

**Table 2:** The age-stratified distribution of insurance status among individuals undergoing cataract surgery.

Variables	Age group				
	70–74 years n (%)	75–79 years n (%)	80–84 years n (%)	85–89 years n (%)	90–100 years n (%)
Overall distribution	22,681 (59.09)	10,357 (26.98)	3914 (10.20)	1165 (3.03)	270 (0.70)
Insurance status—overall	3974 (17.52)	1651 (15.94)	426 (10.88)	99 (8.50%)	20 (7.41%)
Private insurance	3020 (13.32)	1263 (12.19)	308 (7.87)	64 (5.49)	11 (4.07)
Government insurance	954 (4.21)	388 (3.75)	118 (3.01)	35 (3.00)	9 (3.33)
2011–2017 overall distribution	7041 (56.35)	3530 (28.25)	1421 (11.37)	410 (3.28)	94 (0.75)
2011–2017 insurance	750 (10.65)	351 (9.94)	95 (6.69)	20 (4.88)	1 (1.06)
2011–2017 private insurance	718 (10.20)	336 (9.52)	89 (6.26)	18 (4.39)	1 (1.06)
2011–2017 government insurance	32 (0.45)	15 (0.42)	6 (0.42)	2 (0.49)	0 (0)
2018–2022 overall distribution	15,640 (60.41)	6827 (26.37)	2493 (9.63)	755 (2.92)	176 (0.68)
2018–2022 insurance	3224 (20.61)	1300 (19.04)	331 (13.28)	79 (10.46)	19 (10.80)
2018–2022 private insurance	2302 (14.74)	927 (13.58)	219 (8.78)	46 (6.09)	10 (5.68)
2018–2022 government insurance	922 (5.90)	373 (5.46)	112 (4.49)	33 (4.37)	9 (5.11)

The key variables included from the EMRs for analysis were patient demographics, district of residence, socioeconomic status, professional status, ocular diagnosis for each eye, mode of payment, insurance coverage, and visual outcomes after surgery. Patients were also categorised into self-reported socioeconomic groups and occupations (as per standardised inputs in the EMR system during patient registration).^26^ The geographic categorisation of the districts of India was performed using the National Sample Survey Organization (NSSO) system.^27^ The insurance data presented are based on claims made by patients.

### Insurance uptake as an outcome measure

Patients with insurance coverage were classified as having either government insurance or private insurance.^18^ Additionally, a temporal analysis was conducted to present insurance trends by year of patient presentation for the periods 2011–2017 and 2018–2022. As presented in the introduction and Supplementary Fig. S1, the year 2018 marks a temporal divide in insurance regularisation in the country, with the adoption of a nationwide centralised insurance system. Furthermore, even the regional insurance systems appear to span equally before and after 2018. This approach allowed insights into potential shifts in the patterns of insurance coverage over time.

### Statistical analysis

Descriptive statistics, namely, mean, median, SD, IQR, and proportion (data distribution) were employed to characterise the study population, which was divided into five-year age intervals. Frequency data for different variables were constructed using Microsoft Excel 2013. Distribution analyses were performed to compare government and private insurance uptake categories. Temporal trends were explored by comparing patient presentations during 2011–2017 and 2018–2022. Uncorrected visual acuity after the cataract surgery was classified into the following categories based on the WHO classification system: better visual outcomes (visual outcomes better than 6/18 when uncorrected), normal visual outcomes (visual outcomes of 6/18 when uncorrected), and poor visual outcomes (visual outcomes worse than 6/18 when uncorrected). The duration between when surgery was advised, and its actual performance was studied as a time gap for service.

The chi-square square test for independence was employed to test the hypothesis that the independent categorical variable (with and without insurance) is associated with the dependent variable (better visual outcome). The Shapiro–Wilk test was used to assess the normality of the data (days difference between advised and actual surgery). The Mann–Whitney *U* test was employed to evaluate the difference in the time gap between advice and actual surgery of patients who did not utilise insurance uptake versus those who did, as well as between government versus private insurance groups.

Simple and multiple logistic regression techniques were performed on the data with the independent variables (gender, age, district-wise distribution, socio-economic distribution, occupation, systemic diagnosis, and better visual acuity) analysed for the coverage of insurance (a binary variable), with the listed predictors. Odds ratios (OR, adjusted and unadjusted), and their corresponding 95% CI were calculated using R software (v.3.5.1). p values below <0.05 were considered as statistically significant.

### Role of funding source

The funding source had no role in study design, data collection, data analysis, interpretation, or writing of the report.

## Results

The demographic data on the 38,387 patients (45.5% females [17,471]) included in the analysis are presented below. This dataset comprised 41.3% of the total records screened using the study criteria.

### Insurance status

**Fig. 1:**
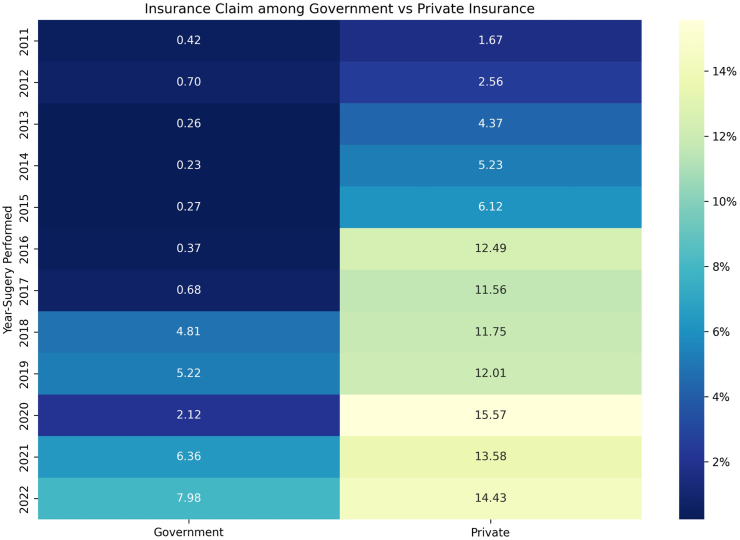
**Graph showing the trend of private versus government insurance uptake for cataract surgical care in the study population on a year-to-year basis. Annual insurance coverage values are plotted from 2011 to 2022**.

**Fig. 2:**
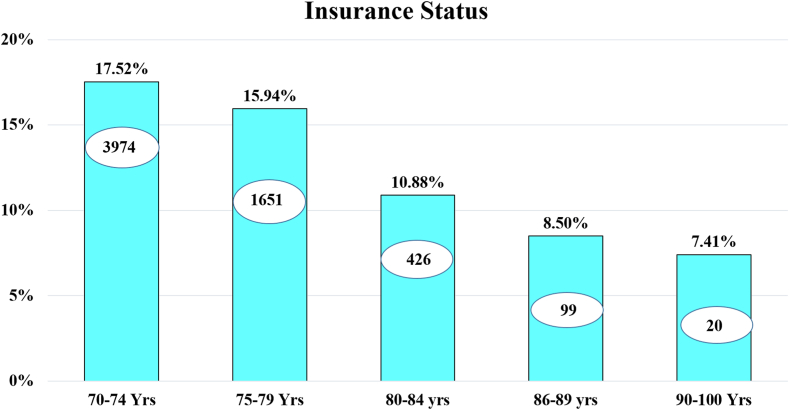
**Bar graph with trendline showing insurance uptake with age groups for cataract surgical care. Age indicated in years (Yrs)**.

**Fig. 3:**
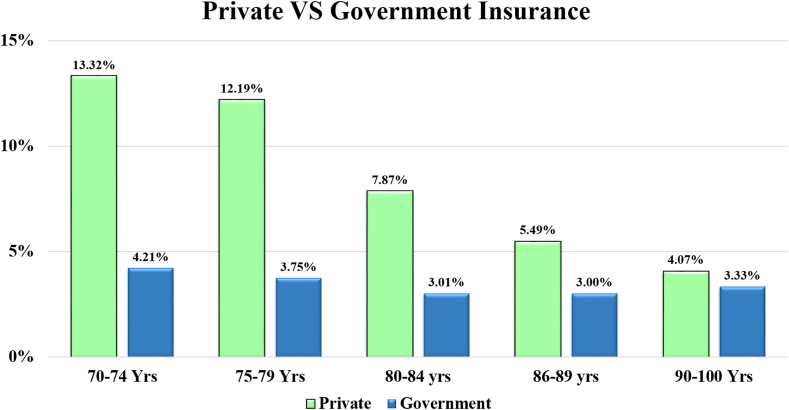
**Composite Bar graph with trendline comparing private versus government insurance uptake across age groups for cataract surgical care. Age indicated in years (Yrs)**.

The baseline data showed that the 70–74 years age group contained the largest number of patients, with declining sample numbers in the older age groups further (Table 1 and Supplementary Table S1). The highest insurance uptake was observed in the age group 70–74 years, with 17.52% (n = 3974) of the patients having insurance uptake. The subsequent age group of 75–79 years exhibited a uptake of 15.94% (n = 1651). In the 80–84 years age group, the insurance uptake was 10.88% (n = 426); in the 85–89 years age group was 8.50% (n = 99); while in the >90 years age group, the insurance uptake was 7.14% (n = 20) for cataract surgery (Table 2) (Fig. 1, Fig. 2, Fig. 3).

### Insurance uptake across socioeconomic status

Overall, 6170 (16.07%) of all patients benefited from insurance uptake. Among these insured patients, 5246 were from the lower middle class, 449 from the upper middle class (UMC), and 286 from the upper class (UC) socioeconomic categories. Overall insurance usage was noted as 14.93% in LMC (5246/35,134), 21.39% in UMC (449/2099), and 29.64% in UC (286/965). The utilisation of government insurance was lower in all groups amongst the insured patients; 25.03% in LMC, 1.11% in UMC, and 0.7% in UC.

### Insurance status across gender

We found insurance uptake to be higher in elderly males compared to elderly females. The overall insurance uptake was 19.11% in males and 12.43% in females (Supplementary Table S2). The uptake remained lower for female across the analysed subgroups of age, socioeconomic status, residence and systemic morbidities (Supplementary Table S2).

### Private and government insurance distribution across age groups

The 70–74 years age group showed 3.17 times more usage of private insurance compared to government insurance (13.32% vs. 4.21%). The 75–79 years age group showed 3.25 times more usage of private insurance compared to government insurance (12.19% vs. 3.75%). For the 80–84 years age group, there was 2.16 times more usage of private insurance compared to government insurance (7.87% vs. 3.01%), and for the 85–90 years age group, 1.83 times more usage of private insurance compared to government insurance (5.49% vs. 3.0%). In the >90 years age group, 1.2 times more usage of private insurance compared to government insurance (4.07% vs. 3.33%) was observed (Table 2). This increasing skew towards government insurance utilisation as compared to private insurance was noted without any proportionate change in the usage of government insurance (3%–4.2%) in all age bands (Fig. 3).

### Temporal trends in government and private insurance

**2011–2017:** During this period, the total insurance uptake was 10.65% (n = 750), with private insurance at 10.20% (n = 718), while government insurance had a minimal presence at 0.45% (n = 32). These findings suggest a substantial reliance on private insurance during the earlier period (Fig. 1).

**2018–2022:** In contrast, the subsequent period, 2018–2022, witnessed a higher insurance uptake of 20.61% (n = 3224). Private insurance continued to exceed government insurance during this period too, with uptake rates 14.74% (n = 2302) and 5.90% (n = 922), respectively. Thus, there was an increased uptake of both, private and government insurance after the 2018 time point, with the latter showing an exponential increase.

### Factors affecting insurance uptake

Simple logistic regression analyses for all the independent variables associated with the dependent variable i.e., insurance uptake, showed multiple statistically significant ORs. The highest OR was for patients from high socioeconomic status (OR = 2.26; p ≤ 0.001, reference–lower middle class) (Table 3), whereas the lowest (and the strongest) was for rural residence (OR: 0.37; p ≤ 0.001, reference–metropolitan residence).

**Table 3:** Factors influencing insurance uptake for cataract surgery in geriatric population.

		Univariate regression			Multivariable regression		
		Unadjusted—OR	95% CI	p-value	Adjusted—OR	95% CI	p-value
Gender ref: female	Male	1.66	1.57–1.76	<0.001	1.71	1.61–1.82	<0.001
Age ref: 81–100 years	70–80 years	1.75	1.65–1.86	<0.001	1.82	1.63–2.03	<0.001
District-wise ref: metropolitan	Rural	0.37	0.35–0.40	<0.001	0.19	0.18–0.21	a<0.001
	Urban	1.21	1.15–1.28	<0.001	0.40	0.37–0.43	
Socio economic ref: lower middle class	Upper middle class	1.45	1.30–1.62	<0.001	1.39	1.24–1.56	a5.616e^−08^
	Upper class	2.26	1.96–2.60	<0.001	1.20	1.03–1.40	
Year—presentation ref: 2011–2017	2018–2022	2.19	2.05–2.34	<0.001	2.65	2.47–2.85	<0.001
Occupation ref: others	Working	1.49	1.33–1.66	<0.001	1.03	0.92–1.16	0.59
Systemic diagnosis ref: no	Yes (diabetes or hypertension)	1.48	1.40–1.57	<0.001	1.54	1.44–1.64	<0.001
Visual acuity ref: worse than 0.3	Last visit UCVA better than 0.3	1.75	1.65–1.86	<0.001	1.43	1.35–1.53	<0.001

OR: Odds; UCVA: Uncorrected visual acuity.

a Global p-value for the category.

**Fig. 4:**
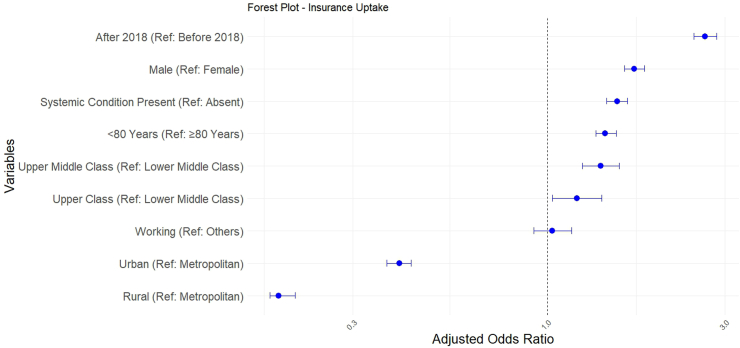
**Forest plot depicting factors affecting insurance uptake in the study population; adjusted odds ratios with 95% CI have been derived through binary logistic regression. Patients operated after 2018 utilised insurance the most; the reference point for socioeconomic status is lower middle class and the reference point for geographical stay is metropolitan city**.

However, the multivariate logistic regression model revealed that patients who had surgeries after 2018 had the highest OR for insurance uptakes (OR = 2.65; CI = 2.4–2.8, p < 0.001, reference–surgery before 2018). This was much higher than all patient-attributable factors including presenting vision, systemic illnesses, gender, age, occupation, and socio-economic status. This signifies the positive impact of the change in regulatory policy for insurance in 2018 (Fig. 4). Rural residence had the lowest association with insurance uptake even in the multivariate regression (OR = 0.18, p ≤ 0.001, reference–metropolitan residence).

### Visual outcomes and insurance uptake

**Fig. 5:**
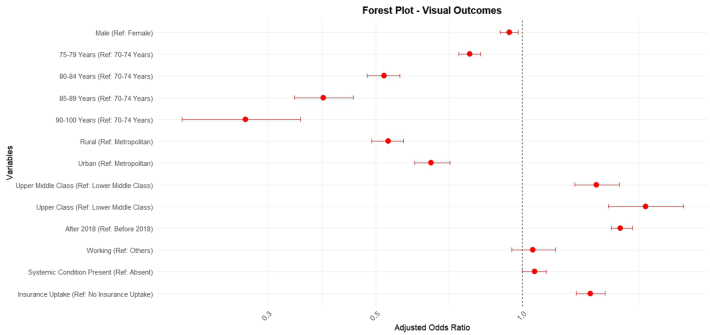
**Forest plot depicting factors associated with good visual outcomes in the study population; adjusted odds ratios with 95% CI have been derived through binary logistic regression; economically better placed individuals and those operated on after 2018 had the best outcomes**.

Good visual outcomes were consistently found to occur more often in individuals using insurance than otherwise across all age groups (Table 4). No useful comparison could be carried out for the >90 years age group, as very few individuals used insurance in that subset. Multivariate regression analysis showed only two factors to be associated with better visual outcomes. The year of presentation after 2018 demonstrated an OR of 1.59, whereas insurance uptake showed the second highest OR of 1.38 (p < 0.001) (Table 5; Fig. 5). This indicates that insured patients are 1.38 times more likely to have better visual outcomes post-surgery than uninsured patients. Higher socio-economic status was also associated with better visual outcomes (OR = 1.42 and 1.79).

**Table 4:** The association between insurance status and good visual outcome (uncorrected visual acuity better than 6/18) stratified by age group.

Good visual outcomes				p-value
Variable (n)	Category (n)	n (%)	Average Log ± SD	
Age 70–74 years (22,586)	With insurance (3961)	2939 (74.20)	0.14 ± 0.11	<0.001
	Without insurance (18,625)	11,527 (61.89)	0.17 ± 0.12	
Age 75–89 years (10,311)	With insurance (1646)	1102 (66.95)	0.16 ± 0.11	<0.001
	Without insurance (8665)	4890 (56.43)	0.17 ± 0.11	
Age 80–84 years (3896)	With insurance (424)	248 (58.49)	0.17 ± 0.10	0.008
	Without insurance (3472)	1623 (46.75)	0.19 ± 0.11	
Age 85–89 years (1156)	With insurance (99)	47 (47.47)	0.17 ± 0.11	0.919
	Without insurance (1060)	421 (39.72)	0.20 ± 0.11	
Age 90–100 years (269)	With insurance (20)	7 (35.00)	0.21 ± 0.09	
	Without insurance (249)	85 (51.83)	0.19 ± 0.11

**Table 5:** Impact of various factors on good visual outcomes (uncorrected visual acuity better than 6/18) in geriatric people undergoing cataract surgery.

		Univariate regression		Multivariable regression		p-value
		Unadjusted—OR	95% CI	Adjusted—OR	95% CI	
Gender ref: female	Male	0.96	0.92–1.01	0.94	0.90–0.98	0.008
Age ref: 70–74 years	75–79 years	0.91	0.87–0.96	0.78	0.74–0.82	a2.621e^−112^
	80–84 years	0.58	0.54–0.62	0.52	0.48–0.56	
	85–89 years	0.47	0.41–0.53	0.39	0.34–0.45	
	90–100 years	0.32	0.24–0.42	0.27	0.20–0.35	
District-wise ref: metropolitan	Rural	0.75	0.71–0.78	0.53	0.49–0.57	a1.025e^−54^
	Urban	1.05	1.00–1.10	0.65	0.60–0.71	
Socio economic ref: lower middle class	Upper middle class	1.34	1.21–1.49	1.42	1.28–1.58	a2.141e^−18^
	Upper class	2.14	1.81–2.55	1.79	1.50–2.14	
Year—presentation ref: 2011–2017	2018–2022	1.59	1.51–1.66	1.59	1.52–1.68	<0.001
Occupation ref: others	Working	1.16	1.05–1.28	1.05	0.95–1.17	0.362
Systemic diagnosis ref: no	Yes (diabetes or hypertension)	1.04	0.99–1.10	1.06	1.00–1.12	0.031
Insurance ref: no	Yes	1.76	1.65–1.88	1.38	1.29–1.48	<0.001

OR: Odds Ratio.

p-values are for Multivariable Regression.

a Global p-value for each category.

### Time gap between surgical advice and surgery

**Supplementary Fig. S2:**
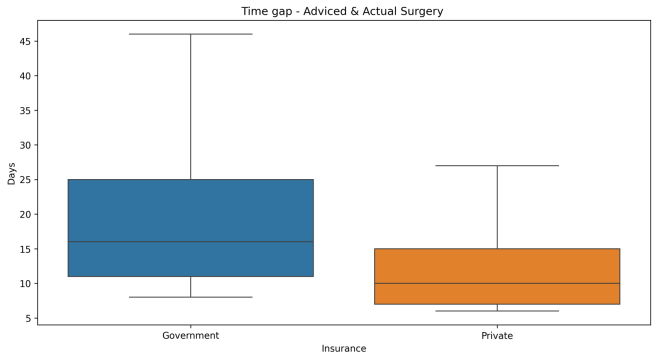
**Box plot depicting the number of days elapsed between when patients were first advised to opt for surgery and the performance of the surgery among those with government and private insurance**.

It took a median gap of 6 days (IQR: 2–16 days) for patients without insurance to have surgery performed after being advised to opt for surgery. In contrast, for those with insurance, this median gap was significantly higher (13 days; IQR: 6–31 days; p < 0.001). Among the latter, 1503 (24.39%) were covered by government insurance; for these patients, it took a median gap of 18 days (IQR: 8–46 days) (p < 0.001) for surgery to be performed after the patients were first advised to opt for surgery. However, for the 4660 (75.61%) patients who were covered by private insurance, the median gap was significantly lower (11 days; IQR: 6–28 days; p < 0.001). The upper bound i.e., 46 days, was substantially higher for government insurance seekers. Government insurance approvals were found to be three times slower than those for private insurance approvals (Supplementary Fig. S2).

### FS conversion to insurance

A total of 189 (0.49%) patients converted from FS to insurance before undergoing surgery, even though they were initially offered the FS service. Nearly all the FS conversions to OOP (n = 183) had surgeries after 2018, and nearly all (n = 184) utilised government insurance. Forty-eight (25.40%) of these patients underwent a planned foldable IOL surgery, thus avoiding a rigid IOL in the case of FS service.

### Types of intraocular lens implants (IOLs)

More elderly males (54.67%) received modern foldable IOLs compared to females (49.18%) (p < 0.001) (Supplementary Table S3). Implantation of foldable IOLs was less common for those over 80 years (44.49% vs. 53.08%, p < 0.001). Higher proportions of elderly people using private insurance received foldable IOLs as compared to those using government insurance (79.55% vs. 53.46%, p < 0.001) (Supplementary Table S3).

### Trends of cataract surgeries with insurance uptake

**Supplementary Fig. S3:**
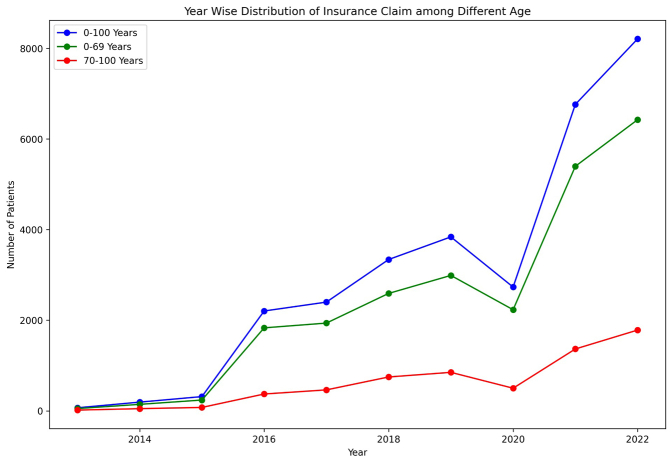
**Annual growth in cataract surgeries with insurance coverage**.

From 2013 to 2022, there was a substantial increase in the number of patients undergoing cataract surgeries with insurance uptake. Starting with 19 surgeries in the baseline year, there was a peak (at 368.92% growth) in 2016, followed by a slight decline in 2020 (likely due to the COVID-19 pandemic), although the overall growth trend remained positive. By 2021, the number of surgeries doubled compared to the previous year, and this growth continued into 2022 with a 42.90% increase. This trend underscores the increasing reliance on and accessibility of insurance for cataract surgeries over the last decade (Supplementary Fig. S3).

## Discussion

In this study, we have described the insurance uptake trends for cataract surgery among 38,387 patients over the age of 70 years. We observed a low uptake of insurance (16.07%) among these patients, with the insurance uptake declining markedly from the ‘youngest old’ (70–74 years) to the ‘oldest old’ (>90 years) (17.52% vs. 7.14%, respectively). Gender disparity was also observed with more proportionate insurance users in elderly males compared to females. The trend was observed across all the subgroups analysed (Supplementary Table S2). This aligns with the findings of Prasad et al., who reported 33% of cataract blindness to be attributable to gender in Indian women.^28^ While the use of private insurance declined with age, government insurance usage remained between 3 and 4%. Patients across all socio-economic strata preferred private insurance over government insurance. The lower-middle income strata showed the highest preference for government insurance (25%). Following the enhancements in the government insurance systems such as *Ayushman Bharat* in 2018, the insurance coverage in patients aged above 70 years doubled to over 20%. This change after 2018 was responsible for not only favouring insurance coverage on regression analysis (OR = 2.65) but was also associated with better visual outcomes (OR = 1.59). The very low insurance uptake for eye care in geriatric subjects reported in this study has also been reported for healthcare in similar populations in other countries (Table 6), including Australia, China Nigeria, and Portugal.^15^^,^^16^^,^^29^, ^30^, ^31^, ^32^, ^33^, ^34^, ^35^, ^36^, ^37^, ^38^, ^39^ This may be due to less awareness,^15^ lesser incentives,^16^ and due to difficulties in navigating through insurance and health care systems faced by the elderly.^35^

**Table 6:** Global trends on insurance utilisation among elderly population for health care.

Authors	Sample size (N), duration of study	Location	Data type	Age criteria	Morbidity type	Insurance utilisation
McWilliams et al.^29^	N = 8736 1 year (1992)	United States	Health and Retirement Study	Near elderly people	Overall Healthcare	82.04%
Wu et al.^30^	N = 69,342 15 years (1993–2018)	China	National Health Service Survey	>60 years	Overall Healthcare	40.1%
Chen et al.^31^	N = 3899 <1995, prior to national health insurance	Taiwan	Surveys of Health and Living status of the Elderly in Taiwan	>60 years	Overall Healthcare	76.69%
Liu et al.^16^	N = 1,612,160 11 years (2001–2012)	Australia	Australian Taxation office (ATO) Longitudinal Information Files	65–69 years 70+ years	Overall Healthcare	35% 40%
Sahoo et al.^32^	N = 72 Studies 20 years (published between 2000 and 2020)	India	Thematic Review	Elderly population	Overall Healthcare	11.5%
Holahan et al.^33^	1 year (2002)	America	National Survey of America's Families	60–64 years	Overall Healthcare	89.9%
Zhang et al.^34^	N = 6690 6 years (2002–2008)	United states	National Health Interview Survey	Above 40 years	Ophthalmic Insurance	96.9%
Tavares et al.^35^ 5701 2014	N = 6690 6 years (2002–2008)	Portugal	National Health Survey	>65 Years	Overall Healthcare	15.17%
Chatterjee et al.^36^	N = 27,225 2 years (2014–2015)	India	71st round of the Unit-level National (NSS)	>60 years	Overall Healthcare	17.77%
Garg et al.^37^ 72,250 2017–2018	N = 72,250 2 years (2017–2018)	India	Longitudinal Ageing Study in India (LASI)	>45 years	Publicly Funded Health Insurance	18.13%
Dai et al.^38^	N = 3871 3 years (2018–2020)	China	Hospital Based Study	>60 years	Mental Illness	21.49%
Stojisavljevic et al.^39^	N = 45 (qualitative) N = 715 (quantitative) 3 months (September–November 2020)	Republic of Srpska's Europe	Mixed Method Study Qualitative–FGD Quantitative–Statistical Office of the Republic of Srpska	>65 years	Community-based Health Insurance	21%
Archibong et al.^15^	N = 358	South Nigeria	Interviewer administered semi-structured questionnaire	>60 years	Community-based Health Insurance	16.5%

The column marked insurance utilisation represents the insurance coverage or uptake reported by the individual research papers.

However, this contrasts with the actual demand for healthcare in the geriatric population. Data from a medical expenditure panel survey from the USA showed that the elderly population constituted around half of the ophthalmic medication users overall.^34^ Trends similar to those reported in this study on eye care expenditure by the elderly are noticeable across other fields, such as mental health, where around a fifth of care seekers over 60 years of age in China depended on OOP spending.^38^ Providing the elderly population access to care through insurance coverage will be important for universal health coverage. A change in policy on rebates for the elderly population in Australia showed that price elasticities for private health insurance are higher for the economically weaker groups.^16^ Taiwan's national health insurance was introduced in 1995. Chen and colleagues reported an increase in utilisation of healthcare systems by the elderly, even for those who had other kinds of insurance available to them (by 14.6%).^31^ However, the authors mention that only the provision of an insurance system could not improve the healthcare metrics in Taiwan, and that continuous reform is needed to make an insurance system useable by the otherwise vulnerable elderly population.^31^ In this lieu, Tavares reported the elderly in Portugal to seek an extra layer of health care protection by demanding voluntary insurance above the otherwise available national health insurance. As per the author, the reason for this demand was the non-availability of effective expert care through the national insurance system.^35^ A similar approach to reform in health policy is needed in the context of India as the newly launched *Ayushman Bharat* scheme (national insurance) gets implemented across the country.^19^

We hypothesised that the incentivising of health insurance uptake for the low-income elderly population can improve access to healthcare. Community-based health insurance is another approach to improving geriatric eye care for this subset of the population.^15^ Socioeconomic disparity still remains a major driver for the usage of age-related eye care in Americans, with lower poverty-to-income ratios creating a barrier to affordable eye care for the elderly population.^34^ Providing accessible medical insurance to economically poor elderly populations can have a poverty reduction effect.^31^

Various government healthcare initiatives worldwide aim to address the comprehensive needs of elderly population eye care. Despite encouraging coverage among the “near-older population” population, eye care for the elderly population remains a complex challenge.^33^ National health insurance programmes have been established by governments to facilitate access to healthcare services, ensuring individuals can receive necessary treatments without financial barriers.^39^ Improving health insurance coverage has been shown to help the elderly population more than the nearly older population by reducing medical expenditure by one-third and family economic burden by nearly 5%.^40^ Unfortunately, however, the situation remains grim in the most populous regions of the world, where the proportion of the elderly population is growing. In China, approximately 29.44% of the elderly population is covered by government employee insurance,^40^ while in India, only 18.13% of the elderly population was enrolled in publicly funded health insurance programs, with government-funded insurance covering only 16% of this population.^36^^,^^37^ This is despite the poverty reduction effects of such schemes in these regions.^40^

Our data underscore the profound influence of having insurance coverage on healthcare outcomes, including visual acuity and surgical timelines. It highlights the critical role of payment systems, including insurance, in driving better visual outcomes for an essential eye care service like cataract surgery, in the elderly population (Fig. 5). As the ‘oldest old’ may be financially dependent on their families, insurance coverage in this vulnerable group may be central to their ocular health. However, our data also revealed concerning disparities in the current situation, with the oldest individuals having limited insurance uptake (7%, Fig. 2). This is explained by the restrictive nature of private insurance schemes which are characterised by high premiums and limitations in coverage leading; this leads to a lower utilisation of private schemes in the oldest population (4%, Fig. 1, Fig. 3). Another issue may be that government schemes are difficult to access for the oldest, as their utilisation remained constant at 3%–4% uptake across all age groups (Fig. 1, Fig. 3). Furthermore, the individuals utilising government schemes had the longest time gap between advice and performance of the surgery. It should be noted here that insurance uptake has shown an increase in the elderly population in our data following 2018 (Fig. 4), when major government schemes were launched with an intention to improve health insurance coverage (Supplementary Fig. S1). Further, people who were offered FS services, switched to government insurance (184 out of 189). This indicates the desire even in those in the FS category to opt for a “paid” care, and their dependence on government schemes for the same. To address this disparity, government interventions are critical, necessitating tailored incentives and policy reforms aimed at enhancing insurance accessibility, easy navigation of insurance formalities, and affordability for elderly adults, thereby ensuring equitable access to vital eye care services. Our data suggest the age group of 80–85 years as the ideal point for implementing such reforms, as at this data point, insurance uptake fell below 10% (Fig. 2). For this population, visual outcomes following cataract surgery worsened with increasing age and parallelled the drop in insurance uptake (Fig. 5).

Our analysis is limited by smaller numbers in the ‘oldest olds’ group which prevented us from drawing certain statistical conclusions. The strength of this study lies in its utilisation of a unique system of eye care where patients using all kinds of payment systems, including OOP, insurance, and FS seek care from one institute. This provides us the opportunity to understand the behavioural patterns in the choice of care, as seen by the conversion of 184 patients from FS to insurance-based care. Further, the data were collected from patients in actual need of eye care at all levels of healthcare pyramid, and not from a data source. We could not analyse the different packages used for availing surgical care by the patients. It is possible that the premium packages may not be available in certain insurance schemes, and this could have affected the choice of payment method used. These limitations notwithstanding, the need to facilitate eye care for the elderly through insurance is critical for India as older age remains significantly associated with visual disability as reported by Vashisht et al., while economic barrier remains a strong determinant of health seeking behaviour by the elderly.^41^^,^^42^

In conclusion, the elderly population, especially individuals aged 80 years and above, need support for eye care. Our study shows that visual outcomes after cataract surgery are better in those with financial support through insurance coverage, apart from the impact of socioeconomic factors. Although governmental insurance reforms have improved care for such individuals, they fall short in filling the gaps that private insurance coverage also fails to fill in this population. Our study demonstrates a critical need for regulatory reforms to expand and incentivise eye care-related insurance coverage across this highly vulnerable age group, which is expected to become a major part of the population in the future.

## Contributors

BT, RV, AVD, and RN contributed to study question, design, and initial analysis. MM, HV, and RN contributed to revised analysis and its interpretations. BT and RV wrote the initial manuscript. BT, RV, and HV created the figures. All authors contributed to revisions and preparation of the final manuscript.

## Data sharing statement

The data is available with the corresponding authors and will be shared on request.

## Declaration of interests

We declare no competing interests.
