# Supplementary material for: Financial vulnerability of the elderly population undergoing cataract surgery within a distributed eye care delivery system in India (2011–2022): a multicentre, retrospective cohort study
Source: Lancet Reg Health Southeast Asia. 2025 Jul 23;39:100640. doi: 10.1016/j.lansea.2025.100640 (PMC12309920; doi:10.1016/j.lansea.2025.100640)
Supplement: Supplementary Tables S1–S3 [file mmc1.docx]

**Supplementary Table: 1 Demographic characteristics of individuals undergoing cataract surgery under insurance cover stratified as per age**

|  | **Variables** | **Age group** | | | | | | | | | |
| --- | --- | --- | --- | --- | --- | --- | --- | --- | --- | --- | --- |
|  |  | **70-74 years** | | **75-79 years** | | **80-84 years** | | **85-89 years** | | **>90 years** | |
|  |  | **Insurance User** | **OOP** | **Insurance User** | **OOP** | **Insurance User** | **OOP** | **Insurance User** | **OOP** | **Insurance User** | **OOP** |
| **Gender** | **Male** | **2526** | **9643** | **1105** | **4701** | **286** | **1872** | **68** | **576** | **13** | **126** |
|  | **Female** | **1448** | **9064** | **546** | **4005** | **140** | **1616** | **31** | **490** | **07** | **124** |
| **District Wise Distribution** | **Urban** | **1670** | **6799** | **727** | **3454** | **176** | **1411** | **41** | **432** | **12** | **123** |
|  | **Rural** | **1195** | **10370** | **476** | **4393** | **126** | **1707** | **29** | **497** | **05** | **106** |
|  | **Metropolitan** | **1109** | **1538** | **448** | **859** | **124** | **370** | **29** | **137** | **03** | **21** |
| **Economic Status** | **Lower Middle class** | **3439** | **17572** | **1369** | **7953** | **345** | **3184** | **81** | **950** | **12** | **229** |
|  | **Upper middle Class** | **264** | **822** | **139** | **552** | **34** | **205** | **09** | **84** | **03** | **17** |
|  | **Upper Class** | **159** | **313** | **90** | **231** | **31** | **99** | **06** | **32** | **0** | **4** |
|  | **Lower Class** | **112** | **0** | **53** | **0** | **16** | **0** | **03** | **0** | **05** | **0** |
| **Systemic Condition** | **Diabetes or Hypertension** | **1348** | **4626** | **561** | **2409** | **141** | **909** | **39** | **297** | **06** | **59** |
| **Occupation** | **Working Professionals** | **324** | **997** | **102** | **424** | **21** | **151** | **04** | **42** | **0** | **7** |

OOP - Out-of-pocket expenditures

| **Supplementary Table 2: Gender variations in payment method used by the elderly population for cataract surgery** | | | | | |
| --- | --- | --- | --- | --- | --- |
|  | **Variables** | **Male – 20916** | | **Female - 17471** | |
|  |  | **Insurance User n (% male)**  **3998 (19.11)** | **OOP**  **n (% male)**  **16918 (80.89)** | **Insurance User n (% female)**  **2172 (12.43)** | **OOP**  **n (% female)**  **15299 (87.57)** |
| **Age** | **70-80 years**  **n = 34334** | **3711 (19.90)** | **14934 (80.10)** | **2040 (13.00)** | **13649 (87.00)** |
|  | **>80 years**  **n = 4053** | **287 (12.64)** | **1984 (87.36)** | **132 (7.41)** | **1650 (92.59)** |
| **District-wise Distribution** | **Rural**  **n = 18904** | **1184 (11.89)** | **8777 (88.11)** | **647 (7.23)** | **8296 (92.77)** |
|  | **Urban**  **n = 14845** | **1734 (20.66)** | **6658 (79.34)** | **892 (13.82)** | **5561 (86.18)** |
|  | **Metropolitan**  **n = 4638** | **1080 (42.14)** | **1483 (57.86)** | **633 (30.51)** | **1442 (69.49)** |
| **Socioeconomic status** | **Lower Middle Class**  **n = 35134** | **3430 (17.92)** | **15715 (82.08)** | **1816 (11.36)** | **14173 (88.64)** |
|  | **Lower Class**  **n = 189** | **135 (100)** | **0 (0)** | **54 (100)** | **0 (0)** |
|  | **Upper Middle Class**  **n = 2099** | **272 (24.24)** | **850 (75.76)** | **177 (18.12)** | **800 (81.88)** |
|  | **Upper Class**  **n = 965** | **161 (31.32)** | **353 (68.68)** | **125 (27.72)** | **326 (72.28)** |
| **Systemic Condition** | **Diabetes or Hypertension**  **n = 10395** | **1332 (23.59)** | **4315 (76.41)** | **763 (16.07)** | **3985 (83.93)** |

| **Supplementary Table 3: Variation in modern foldable vs. traditional implantation of IOLs** | | | | |
| --- | --- | --- | --- | --- |
|  |  | Foldable IOLs – 20028 (73.48) | Non-Foldable IOLs – 18359 (26.52) | P- Value |
| Gender | Male | 11435 (54.67) | 9481 (45.33) | <0.001 |
|  | Female | 8593 (49.18) | 8878 (50.82) |  |
| Age | 70-80 years | 18225 (53.08) | 16109 (46.92) | <0.001 |
|  | >80 years | 1803 (44.49) | 2250 (55.51) |  |
| Insurance | Private | 3712 (79.55) | 954 (20.45) | <0.001 |
|  | Government | 804 (53.46) | 700 (46.54) |  |

IOL: Intraocular lens
